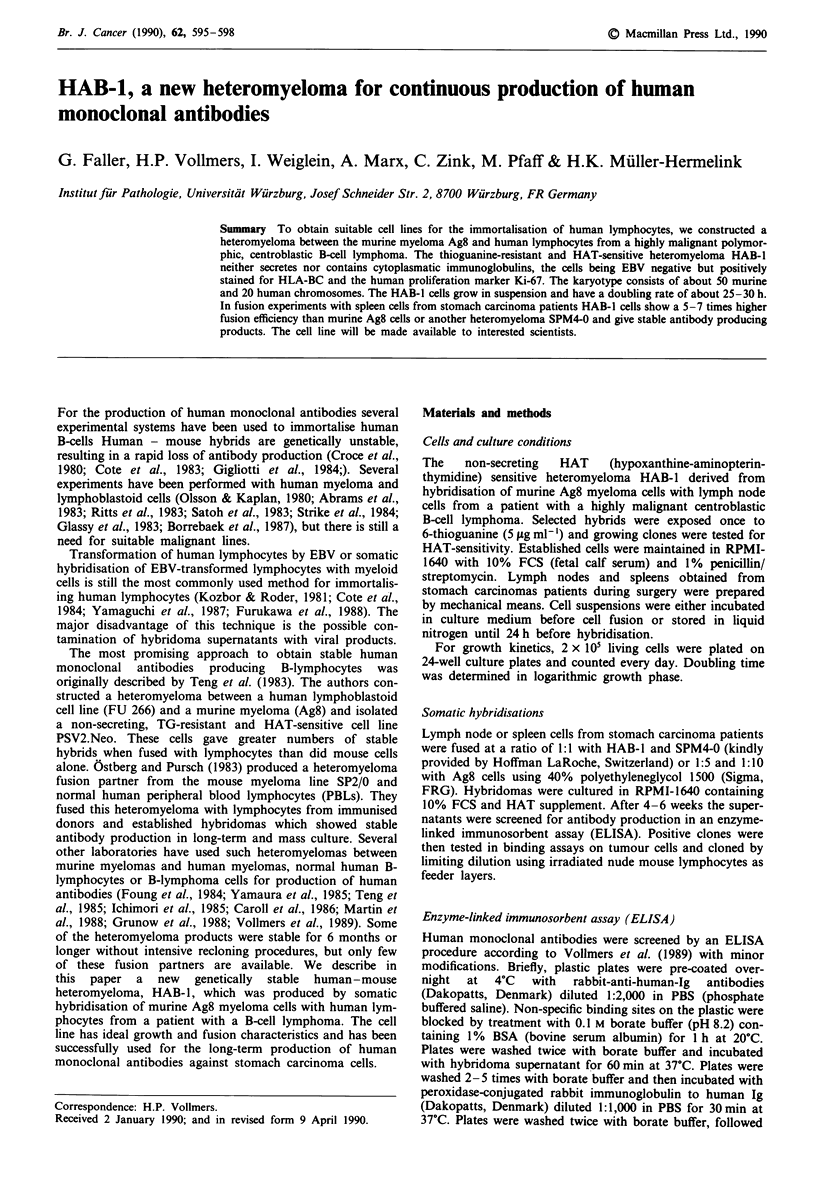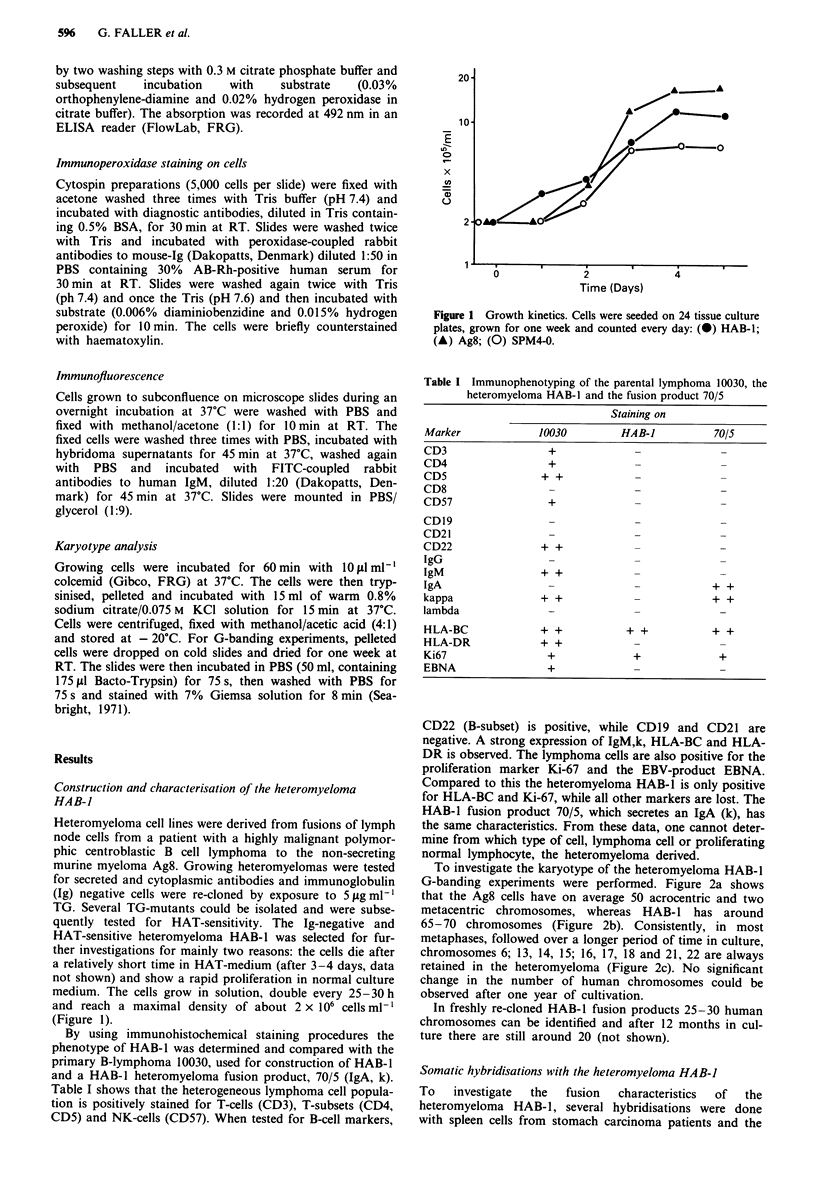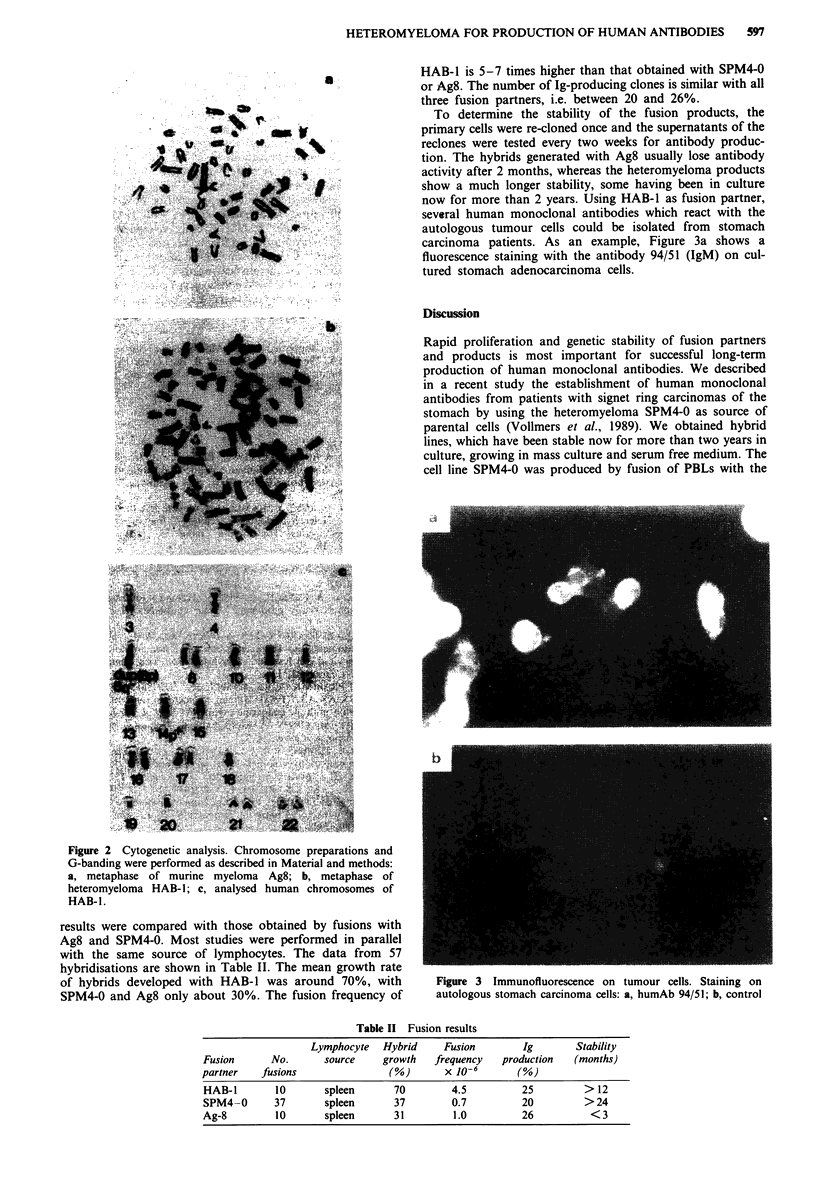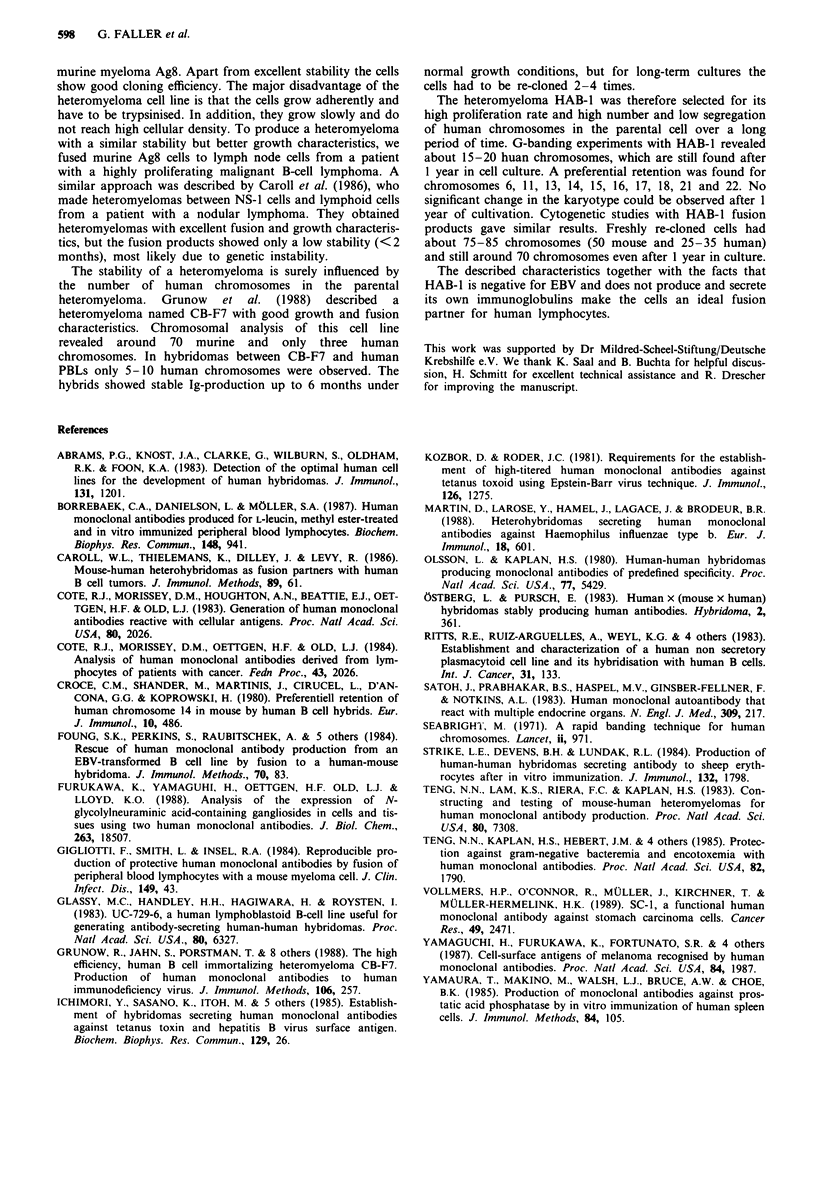# HAB-1, a new heteromyeloma for continuous production of human monoclonal antibodies.

**DOI:** 10.1038/bjc.1990.336

**Published:** 1990-10

**Authors:** G. Faller, H. P. Vollmers, I. Weiglein, A. Marx, C. Zink, M. Pfaff, H. K. Müller-Hermelink

**Affiliations:** Institut für Pathologie, Universität Würzburg, FR Germany.

## Abstract

**Images:**


Br. J. Cancer (1990), 62, 595–598 © Macmillan Press Ltd., 1990

### HAB-1, a new heteromyeloma for continuous production of human monoclonal antibodies

G. Faller, H.P. Vollmers, I. Weiglein, A. Marx, C. Zink, M. Pfaff & H.K. Müller-Hermelink

*Institut für Pathologie, Universität Würzburg, Josef Schneider Str. 2, 8700 Würzburg, FR Germany*

**Summary** To obtain suitable cell lines for the immortalisation of human lymphocytes, we constructed a heteromyeloma between the murine myeloma Ag8 and human lymphocytes from a highly malignant polymorphic, centroblastic B-cell lymphoma. The thioguanine-resistant and HAT-sensitive heteromyeloma HAB-1 neither secretes nor contains cytoplasmatic immunoglobulins, the cells being EBV negative but positively stained for HLA-BC and the human proliferation marker Ki-67. The karyotype consists of about 50 murine and 20 human chromosomes. The HAB-1 cells grow in suspension and have a doubling rate of about 25–30 h. In fusion experiments with spleen cells from stomach carcinoma patients HAB-1 cells show a 5–7 times higher fusion efficiency than murine Ag8 cells or another heteromyeloma SPM4-0 and give stable antibody producing products. The cell line will be made available to interested scientists.

For the production of human monoclonal antibodies several experimental systems have been used to immortalise human B-cells Human – mouse hybrids are genetically unstable, resulting in a rapid loss of antibody production (Croce *et al.*, 1980; Cote *et al.*, 1983; Gigliotti *et al.*, 1984;). Several experiments have been performed with human myeloma and lymphoblastoid cells (Olsson & Kaplan, 1980; Abrams *et al.*, 1983; Ritts *et al.*, 1983; Satoh *et al.*, 1983; Strike *et al.*, 1984; Glassy *et al.*, 1983; Borrebaek *et al.*, 1987), but there is still a need for suitable malignant lines.

Transformation of human lymphocytes by EBV or somatic hybridisation of EBV-transformed lymphocytes with myeloid cells is still the most commonly used method for immortalising human lymphocytes (Kozbor & Roder, 1981; Cote *et al.*, 1984; Yamaguchi *et al.*, 1987; Furukawa *et al.*, 1988). The major disadvantage of this technique is the possible contamination of hybridoma supernatants with viral products.

The most promising approach to obtain stable human monoclonal antibodies producing B-lymphocytes was originally described by Teng *et al.* (1983). The authors constructed a heteromyeloma between a human lymphoblastoid cell line (FU 266) and a murine myeloma (Ag8) and isolated a non-secreting, TG-resistant and HAT-sensitive cell line PSV2.Neo. These cells gave greater numbers of stable hybrids when fused with lymphocytes than did mouse cells alone. Östberg and Pursch (1983) produced a heteromyeloma fusion partner from the mouse myeloma line SP2/0 and normal human peripheral blood lymphocytes (PBLs). They fused this heteromyeloma with lymphocytes from immunised donors and established hybridomas which showed stable antibody production in long-term and mass culture. Several other laboratories have used such heteromyelomas between murine myelomas and human myelomas, normal human B-lymphocytes or B-lymphoma cells for production of human antibodies (Foung *et al.*, 1984; Yamaura *et al.*, 1985; Teng *et al.*, 1985; Ichimori *et al.*, 1985; Caroll *et al.*, 1986; Martin *et al.*, 1988; Grunow *et al.*, 1988; Vollmers *et al.*, 1989). Some of the heteromyeloma products were stable for 6 months or longer without intensive recloning procedures, but only few of these fusion partners are available. We describe in this paper a new genetically stable human–mouse heteromyeloma, HAB-1, which was produced by somatic hybridisation of murine Ag8 myeloma cells with human lymphocytes from a patient with a B-cell lymphoma. The cell line has ideal growth and fusion characteristics and has been successfully used for the long-term production of human monoclonal antibodies against stomach carcinoma cells.

Correspondence: H.P. Vollmers.
Received 2 January 1990; and in revised form 9 April 1990.

### Materials and methods

### *Cells and culture conditions*

The non-secreting HAT (hypoxanthine-aminopterin-thymidine) sensitive heteromyeloma HAB-1 derived from hybridisation of murine Ag8 myeloma cells with lymph node cells from a patient with a highly malignant centroblastic B-cell lymphoma. Selected hybrids were exposed once to 6-thioguanine (5 $\mu g\ ml^{-1}$) and growing clones were tested for HAT-sensitivity. Established cells were maintained in RPMI-1640 with 10% FCS (fetal calf serum) and 1% penicillin/streptomycin. Lymph nodes and spleens obtained from stomach carcinomas patients during surgery were prepared by mechanical means. Cell suspensions were either incubated in culture medium before cell fusion or stored in liquid nitrogen until 24 h before hybridisation.

For growth kinetics, $2 \times 10^5$ living cells were plated on 24-well culture plates and counted every day. Doubling time was determined in logarithmic growth phase.

### *Somatic hybridisations*

Lymph node or spleen cells from stomach carcinoma patients were fused at a ratio of 1:1 with HAB-1 and SPM4-0 (kindly provided by Hoffman LaRoche, Switzerland) or 1:5 and 1:10 with Ag8 cells using 40% polyethyleneglycol 1500 (Sigma, FRG). Hybridomas were cultured in RPMI-1640 containing 10% FCS and HAT supplement. After 4–6 weeks the supernatants were screened for antibody production in an enzyme-linked immunosorbent assay (ELISA). Positive clones were then tested in binding assays on tumour cells and cloned by limiting dilution using irradiated nude mouse lymphocytes as feeder layers.

### *Enzyme-linked immunosorbent assay (ELISA)*

Human monoclonal antibodies were screened by an ELISA procedure according to Vollmers *et al.* (1989) with minor modifications. Briefly, plastic plates were pre-coated overnight at 4°C with rabbit-anti-human-Ig antibodies (Dakopatts, Denmark) diluted 1:2,000 in PBS (phosphate buffered saline). Non-specific binding sites on the plastic were blocked by treatment with 0.1 M borate buffer (pH 8.2) containing 1% BSA (bovine serum albumin) for 1 h at 20°C. Plates were washed twice with borate buffer and incubated with hybridoma supernatant for 60 min at 37°C. Plates were washed 2–5 times with borate buffer and then incubated with peroxidase-conjugated rabbit immunoglobulin to human Ig (Dakopatts, Denmark) diluted 1:1,000 in PBS for 30 min at 37°C. Plates were washed twice with borate buffer, followed by two washing steps with 0.3 M citrate phosphate buffer and subsequent incubation with substrate (0.03% orthophenylene-diamine and 0.02% hydrogen peroxidase in citrate buffer). The absorption was recorded at 492 nm in an ELISA reader (FlowLab, FRG).

*Immunoperoxidase staining on cells*

Cytospin preparations (5,000 cells per slide) were fixed with acetone washed three times with Tris buffer (pH 7.4) and incubated with diagnostic antibodies, diluted in Tris containing 0.5% BSA, for 30 min at RT. Slides were washed twice with Tris and incubated with peroxidase-coupled rabbit antibodies to mouse-Ig (Dakopatts, Denmark) diluted 1:50 in PBS containing 30% AB-Rh-positive human serum for 30 min at RT. Slides were washed again twice with Tris (ph 7.4) and once the Tris (pH 7.6) and then incubated with substrate (0.006% diaminiobenzidine and 0.015% hydrogen peroxide) for 10 min. The cells were briefly counterstained with haematoxylin.

*Immunofluorescence*

Cells grown to subconfluence on microscope slides during an overnight incubation at 37°C were washed with PBS and fixed with methanol/acetone (1:1) for 10 min at RT. The fixed cells were washed three times with PBS, incubated with hybridoma supernatants for 45 min at 37°C, washed again with PBS and incubated with FITC-coupled rabbit antibodies to human IgM, diluted 1:20 (Dakopatts, Denmark) for 45 min at 37°C. Slides were mounted in PBS/glycerol (1:9).

*Karyotype analysis*

Growing cells were incubated for 60 min with 10 μl $ml^{-1}$ colcemid (Gibco, FRG) at 37°C. The cells were then trypsinised, pelleted and incubated with 15 ml of warm 0.8% sodium citrate/0.075 M KCl solution for 15 min at 37°C. Cells were centrifuged, fixed with methanol/acetic acid (4:1) and stored at − 20°C. For G-banding experiments, pelleted cells were dropped on cold slides and dried for one week at RT. The slides were then incubated in PBS (50 ml, containing 175 μl Bacto-Trypsin) for 75 s, then washed with PBS for 75 s and stained with 7% Giemsa solution for 8 min (Seabright, 1971).

### Results

*Construction and characterisation of the heteromyeloma HAB-1*

Heteromyeloma cell lines were derived from fusions of lymph node cells from a patient with a highly malignant polymorphic centroblastic B cell lymphoma to the non-secreting murine myeloma Ag8. Growing heteromyelomas were tested for secreted and cytoplasmic antibodies and immunoglobulin (Ig) negative cells were re-cloned by exposure to 5 μg $ml^{-1}$ TG. Several TG-mutants could be isolated and were subsequently tested for HAT-sensitivity. The Ig-negative and HAT-sensitive heteromyeloma HAB-1 was selected for further investigations for mainly two reasons: the cells die after a relatively short time in HAT-medium (after 3–4 days, data not shown) and show a rapid proliferation in normal culture medium. The cells grow in solution, double every 25–30 h and reach a maximal density of about $2 \times 10^6$ cells $ml^{-1}$ (Figure 1).

By using immunohistochemical staining procedures the phenotype of HAB-1 was determined and compared with the primary B-lymphoma 10030, used for construction of HAB-1 and a HAB-1 heteromyeloma fusion product, 70/5 (IgA, k). Table I shows that the heterogeneous lymphoma cell population is positively stained for T-cells (CD3), T-subsets (CD4, CD5) and NK-cells (CD57). When tested for B-cell markers,

**Figure 1** Growth kinetics. Cells were seeded on 24 tissue culture plates, grown for one week and counted every day: (●) HAB-1; (▲) Ag8; (○) SPM4-0.

**Table I** Immunophenotyping of the parental lymphoma 10030, the heteromyeloma HAB-1 and the fusion product 70/5

| | *Staining on* | | |
|---|---|---|---|
| *Marker* | *10030* | *HAB-1* | *70/5* |
| CD3 | + | − | − |
| CD4 | + | − | − |
| CD5 | + + | − | − |
| CD8 | − | − | − |
| CD57 | + | − | − |
| CD19 | − | − | − |
| CD21 | − | − | − |
| CD22 | + + | − | − |
| IgG | − | − | − |
| IgM | + + | − | − |
| IgA | − | − | + + |
| kappa | + + | − | + + |
| lambda | − | − | − |
| HLA-BC | + + | + + | + + |
| HLA-DR | + + | − | − |
| Ki67 | + | + | + |
| EBNA | + | − | − |

CD22 (B-subset) is positive, while CD19 and CD21 are negative. A strong expression of IgM,k, HLA-BC and HLA-DR is observed. The lymphoma cells are also positive for the proliferation marker Ki-67 and the EBV-product EBNA. Compared to this the heteromyeloma HAB-1 is only positive for HLA-BC and Ki-67, while all other markers are lost. The HAB-1 fusion product 70/5, which secretes an IgA (k), has the same characteristics. From these data, one cannot determine from which type of cell, lymphoma cell or proliferating normal lymphocyte, the heteromyeloma derived.

To investigate the karyotype of the heteromyeloma HAB-1 G-banding experiments were performed. Figure 2a shows that the Ag8 cells have on average 50 acrocentric and two metacentric chromosomes, whereas HAB-1 has around 65–70 chromosomes (Figure 2b). Consistently, in most metaphases, followed over a longer period of time in culture, chromosomes 6; 13, 14, 15; 16, 17, 18 and 21, 22 are always retained in the heteromyeloma (Figure 2c). No significant change in the number of human chromosomes could be observed after one year of cultivation.

In freshly re-cloned HAB-1 fusion products 25–30 human chromosomes can be identified and after 12 months in culture there are still around 20 (not shown).

*Somatic hybridisations with the heteromyeloma HAB-1*

To investigate the fusion characteristics of the heteromyeloma HAB-1, several hybridisations were done with spleen cells from stomach carcinoma patients and the

**Figure 2** Cytogenetic analysis. Chromosome preparations and G-banding were performed as described in Material and methods: **a**, metaphase of murine myeloma Ag8; **b**, metaphase of heteromyeloma HAB-1; **c**, analysed human chromosomes of HAB-1.

results were compared with those obtained by fusions with Ag8 and SPM4-0. Most studies were performed in parallel with the same source of lymphocytes. The data from 57 hybridisations are shown in Table II. The mean growth rate of hybrids developed with HAB-1 was around 70%, with SPM4-0 and Ag8 only about 30%. The fusion frequency of HAB-1 is 5–7 times higher than that obtained with SPM4-0 or Ag8. The number of Ig-producing clones is similar with all three fusion partners, i.e. between 20 and 26%.

To determine the stability of the fusion products, the primary cells were re-cloned once and the supernatants of the reclones were tested every two weeks for antibody production. The hybrids generated with Ag8 usually lose antibody activity after 2 months, whereas the heteromyeloma products show a much longer stability, some having been in culture now for more than 2 years. Using HAB-1 as fusion partner, several human monoclonal antibodies which react with the autologous tumour cells could be isolated from stomach carcinoma patients. As an example, Figure 3a shows a fluorescence staining with the antibody 94/51 (IgM) on cultured stomach adenocarcinoma cells.

### Discussion

Rapid proliferation and genetic stability of fusion partners and products is most important for successful long-term production of human monoclonal antibodies. We described in a recent study the establishment of human monoclonal antibodies from patients with signet ring carcinomas of the stomach by using the heteromyeloma SPM4-0 as source of parental cells (Vollmers *et al.*, 1989). We obtained hybrid lines, which have been stable now for more than two years in culture, growing in mass culture and serum free medium. The cell line SPM4-0 was produced by fusion of PBLs with the

**Figure 3** Immunofluorescence on tumour cells. Staining on autologous stomach carcinoma cells: **a**, humAb 94/51; **b**, control

**Table II** Fusion results

| Fusion partner | No. fusions | Lymphocyte source | Hybrid growth (%) | Fusion frequency × $10^{-6}$ | Ig production (%) | Stability (months) |
|---|---|---|---|---|---|---|
| HAB-1 | 10 | spleen | 70 | 4.5 | 25 | >12 |
| SPM4–0 | 37 | spleen | 37 | 0.7 | 20 | >24 |
| Ag-8 | 10 | spleen | 31 | 1.0 | 26 | <3 |

murine myeloma Ag8. Apart from excellent stability the cells show good cloning efficiency. The major disadvantage of the heteromyeloma cell line is that the cells grow adherently and have to be trypsinised. In addition, they grow slowly and do not reach high cellular density. To produce a heteromyeloma with a similar stability but better growth characteristics, we fused murine Ag8 cells to lymph node cells from a patient with a highly proliferating malignant B-cell lymphoma. A similar approach was described by Caroll *et al.* (1986), who made heteromyelomas between NS-1 cells and lymphoid cells from a patient with a nodular lymphoma. They obtained heteromyelomas with excellent fusion and growth characteristics, but the fusion products showed only a low stability (<2 months), most likely due to genetic instability.

The stability of a heteromyeloma is surely influenced by the number of human chromosomes in the parental heteromyeloma. Grunow *et al.* (1988) described a heteromyeloma named CB-F7 with good growth and fusion characteristics. Chromosomal analysis of this cell line revealed around 70 murine and only three human chromosomes. In hybridomas between CB-F7 and human PBLs only 5–10 human chromosomes were observed. The hybrids showed stable Ig-production up to 6 months under normal growth conditions, but for long-term cultures the cells had to be re-cloned 2–4 times.

The heteromyeloma HAB-1 was therefore selected for its high proliferation rate and high number and low segregation of human chromosomes in the parental cell over a long period of time. G-banding experiments with HAB-1 revealed about 15–20 huan chromosomes, which are still found after 1 year in cell culture. A preferential retention was found for chromosomes 6, 11, 13, 14, 15, 16, 17, 18, 21 and 22. No significant change in the karyotype could be observed after 1 year of cultivation. Cytogenetic studies with HAB-1 fusion products gave similar results. Freshly re-cloned cells had about 75–85 chromosomes (50 mouse and 25–35 human) and still around 70 chromosomes even after 1 year in culture.

The described characteristics together with the facts that HAB-1 is negative for EBV and does not produce and secrete its own immunoglobulins make the cells an ideal fusion partner for human lymphocytes.

This work was supported by Dr Mildred-Scheel-Stiftung/Deutsche Krebshilfe e.V. We thank K. Saal and B. Buchta for helpful discussion, H. Schmitt for excellent technical assistance and R. Drescher for improving the manuscript.

### References

ABRAMS, P.G., KNOST, J.A., CLARKE, G., WILBURN, S., OLDHAM, R.K. & FOON, K.A. (1983). Detection of the optimal human cell lines for the development of human hybridomas. *J. Immunol.*, **131**, 1201.

BORREBAEK, C.A., DANIELSON, L. & MÖLLER, S.A. (1987). Human monoclonal antibodies produced for L-leucin, methyl ester-treated and in vitro immunized peripheral blood lymphocytes. *Biochem. Biophys. Res. Commun.*, **148**, 941.

CAROLL, W.L., THIELEMANS, K., DILLEY, J. & LEVY, R. (1986). Mouse-human heterohybridomas as fusion partners with human B cell tumors. *J. Immunol. Methods*, **89**, 61.

COTE, R.J., MORISSEY, D.M., HOUGHTON, A.N., BEATTIE, E.J., OETTGEN, H.F. & OLD, L.J. (1983). Generation of human monoclonal antibodies reactive with cellular antigens. *Proc. Natl Acad. Sci. USA*, **80**, 2026.

COTE, R.J., MORISSEY, D.M., OETTGEN, H.F. & OLD, L.J. (1984). Analysis of human monoclonal antibodies derived from lymphocytes of patients with cancer. *Fedn Proc.*, **43**, 2026.

CROCE, C.M., SHANDER, M., MARTINIS, J., CIRUCEL, L., D'ANCONA, G.G. & KOPROWSKI, H. (1980). Preferentiell retention of human chromosome 14 in mouse by human B cell hybrids. *Eur. J. Immunol.*, **10**, 486.

FOUNG, S.K., PERKINS, S., RAUBITSCHEK, A. & 5 others (1984). Rescue of human monoclonal antibody production from an EBV-transformed B cell line by fusion to a human-mouse hybridoma. *J. Immunol. Methods.*, **70**, 83.

FURUKAWA, K., YAMAGUHI, H., OETTGEN, H.F. OLD, L.J. & LLOYD, K.O. (1988). Analysis of the expression of *N*-glycolylneuraminic acid-containing gangliosides in cells and tissues using two human monoclonal antibodies. *J. Biol. Chem.*, **263**, 18507.

GIGLIOTTI, F., SMITH, L. & INSEL, R.A. (1984). Reproducible production of protective human monoclonal antibodies by fusion of peripheral blood lymphocytes with a mouse myeloma cell. *J. Clin. Infect. Dis.*, **149**, 43.

GLASSY, M.C., HANDLEY, H.H., HAGIWARA, H. & ROYSTEN, I. (1983). UC-729-6, a human lymphoblastoid B-cell line useful for generating antibody-secreting human-human hybridomas. *Proc. Natl Acad. Sci. USA.*, **80**, 6327.

GRUNOW, R., JAHN, S., PORSTMAN, T. & 8 others (1988). The high efficiency, human B cell immortalizing heteromyeloma CB-F7. Production of human monoclonal antibodies to human immunodeficiency virus. *J. Immunol. Methods*, **106**, 257.

ICHIMORI, Y., SASANO, K., ITOH, M. & 5 others (1985). Establishment of hybridomas secreting human monoclonal antibodies against tetanus toxin and hepatitis B virus surface antigen. *Biochem. Biophys. Res. Commun.*, **129**, 26.

KOZBOR, D. & RODER, J.C. (1981). Requirements for the establishment of high-titered human monoclonal antibodies against tetanus toxoid using Epstein-Barr virus technique. *J. Immunol.*, **126**, 1275.

MARTIN, D., LAROSE, Y., HAMEL, J., LAGACE, J. & BRODEUR, B.R. (1988). Heterohybridomas secreting human monoclonal antibodies against Haemophilus influenzae type b. *Eur. J. Immunol.*, **18**, 601.

OLSSON, L. & KAPLAN, H.S. (1980). Human-human hybridomas producing monoclonal antibodies of predefined specificity. *Proc. Natl Acad. Sci. USA.*, **77**, 5429.

ÖSTBERG, L. & PURSCH, E. (1983). Human × (mouse × human) hybridomas stably producing human antibodies. *Hybridoma*, **2**, 361.

RITTS, R.E., RUIZ-ARGUELLES, A., WEYL, K.G. & 4 others (1983). Establishment and characterization of a human non secretory plasmacytoid cell line and its hybridisation with human B cells. *Int. J. Cancer*, **31**, 133.

SATOH, J., PRABHAKAR, B.S., HASPEL, M.V., GINSBER-FELLNER, F. & NOTKINS, A.L. (1983). Human monoclonal autoantibody that react with multiple endocrine organs. *N. Engl. J. Med.*, **309**, 217.

SEABRIGHT, M. (1971). A rapid banding technique for human chromosomes. *Lancet*, **ii**, 971.

STRIKE, L.E., DEVENS, B.H. & LUNDAK, R.L. (1984). Production of human-human hybridomas secreting antibody to sheep erythrocytes after in vitro immunization. *J. Immunol.*, **132**, 1798.

TENG, N.N., LAM, K.S., RIERA, F.C. & KAPLAN, H.S. (1983). Constructing and testing of mouse-human heteromyelomas for human monoclonal antibody production. *Proc. Natl Acad. Sci. USA*, **80**, 7308.

TENG, N.N., KAPLAN, H.S., HEBERT, J.M. & 4 others (1985). Protection against gram-negative bacteremia and encotoxemia with human monoclonal antibodies. *Proc. Natl Acad. Sci. USA*, **82**, 1790.

VOLLMERS, H.P., O'CONNOR, R., MÜLLER, J., KIRCHNER, T. & MÜLLER-HERMELINK, H.K. (1989). SC-1, a functional human monoclonal antibody against stomach carcinoma cells. *Cancer Res.*, **49**, 2471.

YAMAGUCHI, H., FURUKAWA, K., FORTUNATO, S.R. & 4 others (1987). Cell-surface antigens of melanoma recognised by human monoclonal antibodies. *Proc. Natl Acad. Sci. USA*, **84**, 1987.

YAMAURA, T., MAKINO, M., WALSH, L.J., BRUCE, A.W. & CHOE, B.K. (1985). Production of monoclonal antibodies against prostatic acid phosphatase by in vitro immunization of human spleen cells. *J. Immunol. Methods*, **84**, 105.